# Neural precursor cells form integrated brain-like tissue when implanted into rat cerebrospinal fluid

**DOI:** 10.1038/s42003-018-0113-8

**Published:** 2018-08-14

**Authors:** Nikorn Pothayee, Dragan Maric, Kathryn Sharer, Jung-Hwa Tao-Cheng, Alec Calac, Nadia Bouraoud, James Pickel, Stephen Dodd, Alan Koretsky

**Affiliations:** 1 Laboratory of Functional and Molecular Imaging, National Institute of Neurological Disorders and Stroke, National Institutes of Health, Bethesda, MD 20892 USA; 2 Flow and Imaging Cytometry Core Facility, National Institute of Neurological Disorders and Stroke, National Institutes of Health, Bethesda, MD 20892 USA; 3 Electron Microscopy Facility, National Institute of Neurological Disorders and Stroke, National Institutes of Health, Bethesda, MD 20892 USA; 4 Transgenic Core Facility, National Institute of Mental Health, National Institutes of Health, Bethesda, MD 20892 USA

## Abstract

There is tremendous interest in transplanting neural precursor cells for brain tissue regeneration. However, it remains unclear whether a vascularized and integrated complex neural tissue can be generated within the brain through transplantation of cells. Here, we report that early stage neural precursor cells recapitulate their seminal properties and develop into large brain-like tissue when implanted into the rat brain ventricle. Whereas the implanted cells predominantly differentiated into glutamatergic neurons and astrocytes, the host brain supplied the intact vasculature, oligodendrocytes, GABAergic interneurons, and microglia that seamlessly integrated into the new tissue. Furthermore, local and long-range axonal connections formed mature synapses between the host brain and the graft. Implantation of precursor cells into the CSF-filled cavity also led to a formation of brain-like tissue that integrated into the host cortex. These results may constitute the basis of future brain tissue replacement strategies.

## Abstract (web summary)

Nikorn Pothayee et al. show that early neural precursor cells (NPCs) derived from the embryonic telencephalon or midbrain can develop into brain-like tissue when implanted into the rat brain ventricle. Telencephalon-derived NPCs also form brain tissue in the host cortex when implanted into a CSF-filled cavity formed by cortical ablation.

## Introduction

Progress in neural stem cell research has advanced so that complex neural tissue structures, or organoids, can be formed in vitro^1,2^. These 3-dimensional (3D) organoids have provided a tool for understanding central nervous system (CNS) development and disease mechanisms^3,4^. While much work has been done to integrate new cell grafts into the brain, there have been fewer efforts to form complex neural tissue in vivo, which may be necessary for the repair of brain injury and treatment of neurological diseases^5^. Given the tremendous interest in brain tissue repair, we explored whether large complex brain tissue could develop within an adult brain. The cerebrospinal fluid (CSF) serves as an important niche and is crucial to maintain proliferative activity and differentiation of early neural precursor cells throughout neocortical development and in adulthood^6,7^. Therefore, we tested whether CSF in the brain can support the proliferation and differentiation of implanted neural precursor cells and enable them to form complex brain tissue structures.

Our experiments demonstrate that implanting early cortical neural precursor cells into the CSF space of the rat brain led to a remarkable proliferation and differentiation of precursor cells, forming large brain-like tissues that seamlessly attached and integrated with the host brain, without induction of glial scarring or eliciting an inflammatory response or graft rejection by the host. The tissue structures were extensively vascularized by blood vessels that had an intact blood–brain barrier (BBB) from the host brain. There was large-scale migration of oligodendrocytes, GABAergic neurons, and microglia from the host into the new tissues from the host. Integration with the host brain was evidenced by rearrangement of the host ependymal layer at the graft-host interface and the presence of neural processes between the host and the new tissues. Finally, we show that brain-like tissue derived from cortical precursor cells could develop within a CSF-filled, injury-induced cavity in the cortex of adult rats and seamlessly integrated into the host brain, through the elimination of the glial scar, while also generating extensive long-range axonal projections throughout the host brain.

## Results

### Early neural precursor cells formed brain-like tissues in the CSF

**Fig. 1:**
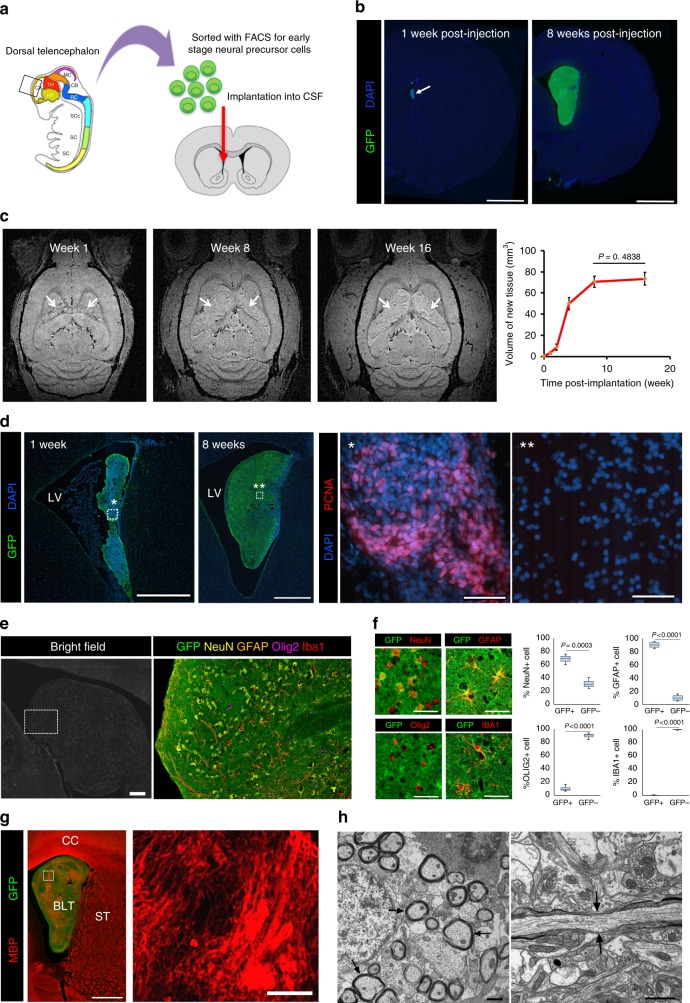
Formation of intraventricular brain-like tissue following implantation of sorted E14 telencephalon neural precursor cells into the lateral ventricles of 3-week old rats. **a** Location of embryonic telencephalic tissue (boxed area) from where the neural precursors were micro dissected and sorted, and the site of intraventricular injection of these cells into the CSF (red arrow). **b** Formation of intra-CSF tissue at 1 week and 8 weeks post-implantation. Scale bar = 2 mm. **c** MRI imaging showed no increase in tissue sizes after 8 weeks. Data represent mean ± standard deviation, *n* = 4. **d** Immunostaining for PCNA showed high proliferation activity of PCNA at the early phase (*) of the tissue growth but not at later stage (**), in line with growth kinetic measured from MRI. Asterisks in the left two panels mark the areas of the enlarged images on right. Scale bars = 500, 1000, 50, 50 μm from left to right panels, respectively. **e** Bright field and fluorescent images showed that the new tissue contained cell types typical of the brain parenchyma including neurons (NeuN), astrocytes (GFAP), oligodendrocytes (Olig2), and microglia (Iba1). Scale bar = 250 μm. **f** High magnification images and quantitation analysis indicate that the bulk of neurons and astrocytes were of implant origin (GFP-positive), and the bulk of oligodendrocytes and all of microglia were of host origin (GFP-negative). Scale bars = 50 μm. **g** Immunostaining for MBP showed presence of myelin throughout the new tissue. Scale bars = 1 mm and 100 μm from left to right. **h** Electron micrographs showed bundles of myelinated axons (arrows, left panel) within the new tissue and node of Ranvier-like structures along the myelinated axons (arrows, right panel). Scale bars = 1 μm

Prior to the implantation, green fluorescent protein (GFP)-positive neural precursor cells lacking the surface phenotyping markers expressed by early glial or neuronal progenitors and their post-mitotic counterparts, as well as lacking markers expressed by resident non-neural cells, were isolated and enriched from E14 rat dorsal telencephalon dissociates using fluorescence activated cell sorting (FACS), as previously described^8^ (Fig. 1a). This method allowed for the removal of any lineage-committed GFP-positive, neuronal and glial cell phenotypes, as well as microglia, endothelial cells, and pericytes residing in the parenchyma of E14 telencephalon, from an uncommitted GFP-positive and lineage-negative (Lin-) population that itself exhibited both self-renewing and multi-potential seminal properties characteristic of neural stem/precursor cells^8^. Approximately 2.5 × 10^5^ Lin(−) cells were injected into the lateral ventricle of 3-week old rats through the right hemisphere. One week following implantation, there were small clusters of mostly undifferentiated cells along the ventricular wall and by 8 weeks, the clusters had greatly expanded into a mass occupying the ventricles (Fig. 1b). None of the rats (*n* = 8) experienced signs of distress from implantation and survived until the time of euthanization. In a separate set of animals (*n* = 4), the growth kinetics of new tissue was monitored in vivo by magnetic resonance imaging (MRI). From MRI images, the implanted cells spread throughout the ventricle and formed various tissue masses in both hemispheres, which is most likely brought on by the flow of CSF (Fig. 1c, Supplementary Movie 1). The implant showed rapid growth during the first four weeks followed by a slowing of the growth rate between week four and week eight, after which the tissue showed no significant further increase in size (Fig. 1c) (*P* = 0.4838, unpaired *t*-test, *t* = 0.7461). The total tissue volume estimated from MRI was 74 ± 6 mm^3^, which is 18–20% of the volume of adult rat cerebral cortex^9^. A cell dose experiment to determine a minimum cell number to initiate this process was performed using 2.5 × 10^4^, 5 × 10^4^, 1.25 × 10^5^, and 2.5 × 10^5^ cells (*n* = 3) showing that at least 5 × 10^4^ Lin(−) cells were needed to form tissue-like structures in the ventricle (Supplementary Fig. 1). Immunostaining for PCNA expression, a proliferation marker, showed that proliferation was high at 1 week post-implantation (* in Fig. 1d), but subsided with time yielding few to non detectable PCNA(+) cells from the implant-derived tissue at 8 weeks post-implantation (** in Fig. 1d).

Cell phenotypes were identified within the brain-like tissue using immunohistochemistry and demonstrated all four of the major types of brain cells including neurons, astrocytes, oligodendrocytes, and microglia (Fig. 1e). Immunostaining with appropriate markers (pan-cytokeratin, alpha-fetoprotein, smooth muscle actin)^10^ indicated that the implant-derived tissue was not a teratoma and that the engrafted cells remained within the neural ectodermal lineage (Supplementary Fig. 2). The origin of each cell type was distinguished using GFP as a marker of the implanted cells whereas cells lacking GFP were identified as originating from the host brain (Fig. 1f). Examination of tissues showed that cells derived from the implanted neural precursors differentiated mainly into neurons and astrocytes. Approximately 70% of the neuron population in the implant were derived from the precursor cells (GFP-positive) and all of these neurons were identified as glutaminase-positive indicating that there were glutamatergic neurons, as expected^8^ (Supplementary Fig. 3). There was evidence that host-derived neurons migrated into the new tissue that located in the lateral ventricles near the host subventricular zone (SVZ). It was found that up to 30% of neuron population in the implant were from host (NeuN-positive and GFP-negative cells). Astrocyte population was mainly derived from implanted precursor cells with approximately 90% of astrocytes expressing GFP (Fig. 1f). These cells were confirmed as mature astrocytes by co-immunostaining with S100B and GFAP (Supplementary Fig. 4). Interestingly, more than 90% of the oligodendrocytes in the transplant were derived from the host (Fig. 1f). Whereas the aforementioned brain cell phenotypes could be contributed by both host and the implanted cells, microglia were found to only originate from host (all were GFP-negative) (Fig. 1f). These microglia within the new tissue had the ramified morphology characteristic of resting microglia and did not express ED1 (CD68), a marker for activated microglia^11^ (Supplementary Fig. 5).

The presence of oligodendrocytes suggests that they were recruited to myelinate axons within the new tissue. Using an antibody against myelin basic protein (MBP), we found MBP-labeled fibers throughout the tissue (Fig. 1g). They were found in distinct MBP-positive regions, one type was characterized by sparse myelination forming tract-like structures associated with neurons and another region that consisted of dense myelinated bundles containing few neuronal nuclei (Supplementary Fig. 6). Ultrastructural characterization by electron microscopy (EM) confirmed an extensive distribution of myelinated fibers with node of Ranvier-like structures implying mature myelination (Fig. 1h).

### Vascularization and integration into the host brain

**Fig. 2:**
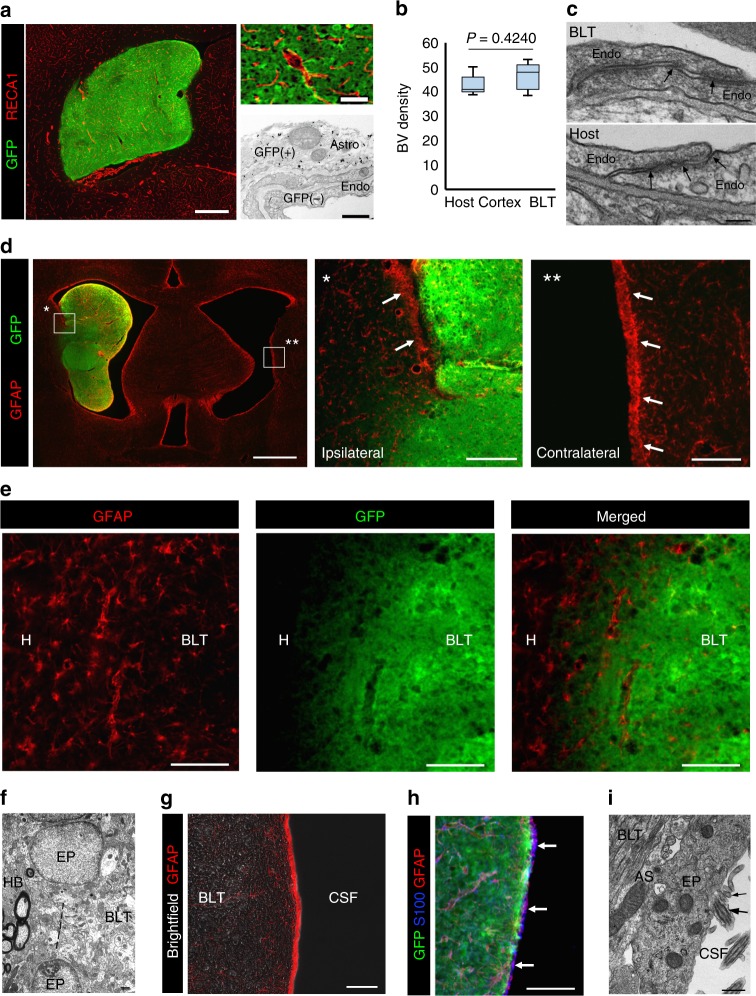
Vascularization and integration into the host brain parenchyma. **a** Immunostaining for RECA1 showed vascularization of new tissues with blood vessels, IHC and EM confirmed that the vascular endothelium (Endo) originated from the host (GFP-negative) and peri-endothelial astrocytes (Astro) were derived from the implanted cells (GFP-positive). Scale bars = 1 mm, 50 μm, and 0.5 μm from left to right. **b** Vessel density in the new tissue was similar to those found in the host cortex (*P* = 0.424, *t* = 0.8425). **c** EM assessment of the endothelial cells lining the lumen of a blood vessel forming tight junctions (TJ, marked by arrows) characteristic of the BBB which is similar to the TJ examined from the host cortex (lower image). Scale bar = 0.1 μm. **d** Immunostaining with anti-GFAP showed the area where the new tissue fused with the host brain displace the GFAP-layer Scale bar = 1 mm, 200 μm, 200 μm from left to right. **e** There was no glial scar between the interface of the new tissue and host brain. Scale bar = 100 μm. **f** Electron micrograph demonstrating the fusion of the new tissue (BLT on right) with the host brain (HB on left) through breaking (dashed line) of the ependymal layer (EP on top and bottom). Scale bar = 0.5 µm. **g** Astrocytic layer formed barriers that isolated the new formed tissue from the CSF. Scale bar = 50 μm. **h** Immunostaining for GFAP and S100 showed that the tissue-CSF interfaces were isolated by GFAP-positive cells and host ependymal cells (S100-positive). Scale bar = 100 μm. **i** EM confirmed the presence of ependymal cells (EP) with characteristic cilias (large arrow) and microvilli (small arrow) atop of astrocytes (AS) at the border between the new tissue (BLT) and CSF. Scale bar = 0.1 µm

Immunostaining against rat endothelial cell antigen (RECA-1), a marker for vascular endothelium, revealed that the new tissue was well-vascularized (Fig. 2a). IHC and EM results showed that the endothelial cells of the vasculature did not express GFP, indicating they were exclusively derived from the host (Fig. 2a). Notably, the blood vessels of the new tissue were similar in density and morphology when compared to those of the host (Fig. 2b) (*P* = 0.424, unpaired *t*-test, *t* = 0.8425) and displayed a normal BBB tight junctions (Fig. 2c) and intact vasculature (Supplementary Fig. 7).

Examination of the interface between the host and grafts showed that the tissue not only attached to the ventricle wall but also integrated with the host without a glial scar and led to remodeling of the host SVZ along lateral ventricle (Fig. 2d, e, Supplementary Fig. 8). Interestingly, the ependymal layer between the host and the attached new tissue were disrupted and this presumably allowed the grafted cells to integrate and fuse into the host (Fig. 2f). At the interface of the new tissue and CSF, immunolabeling of GFAP indicated that there was an astrocyte layer covering the new tissue (Fig. 2g). Further immunostaining with S100 identified host ependymal layer in this area (Fig. 2h), the identity of which was confirmed by EM ultrastructural characterization (Fig. 2i).

Indeed, this lack of astroglia boundary at the tissue-host interface and similar MRI contrast of the new tissue with respect to normal brain further distinguished the new tissue from benign brain tumors that resulted from transplanted fetal stem cells or those observed in humans^12^. Furthermore, despite the large size of the new tissue, none of the rats exhibited signs of adverse effects. They grew normally compared with control rats (Supplementary Fig. 9A). For long-term assessment, a group of rats (*n* = 4) were implanted with neural precursor cells and kept beyond 12 months with the new tissues remained intact (Supplementary Fig. 9B). They showed no sign of distress or deficit (Supplementary Movie 2) and no evidence of neurological symptoms.

To further test that newly-formed tissue from implanted cells into the CSF presented a mature brain phenotype that maintained its developmental lineage and regional identity, we tested whether other precursor cells from additional brain regions could develop into integrated tissue. To this end, early neural precursors from midbrain were isolated and implanted into the ventricles of young adult rats. Indeed, the midbrain precursors developed into brain-like tissue that had phenotypes of the midbrain and were distinctive from the cortical-derived tissue (Supplementary Fig. 10, Supplementary Note 1).

### Brain-like tissues interact with host subventricular zone (SVZ)

**Fig. 3:**
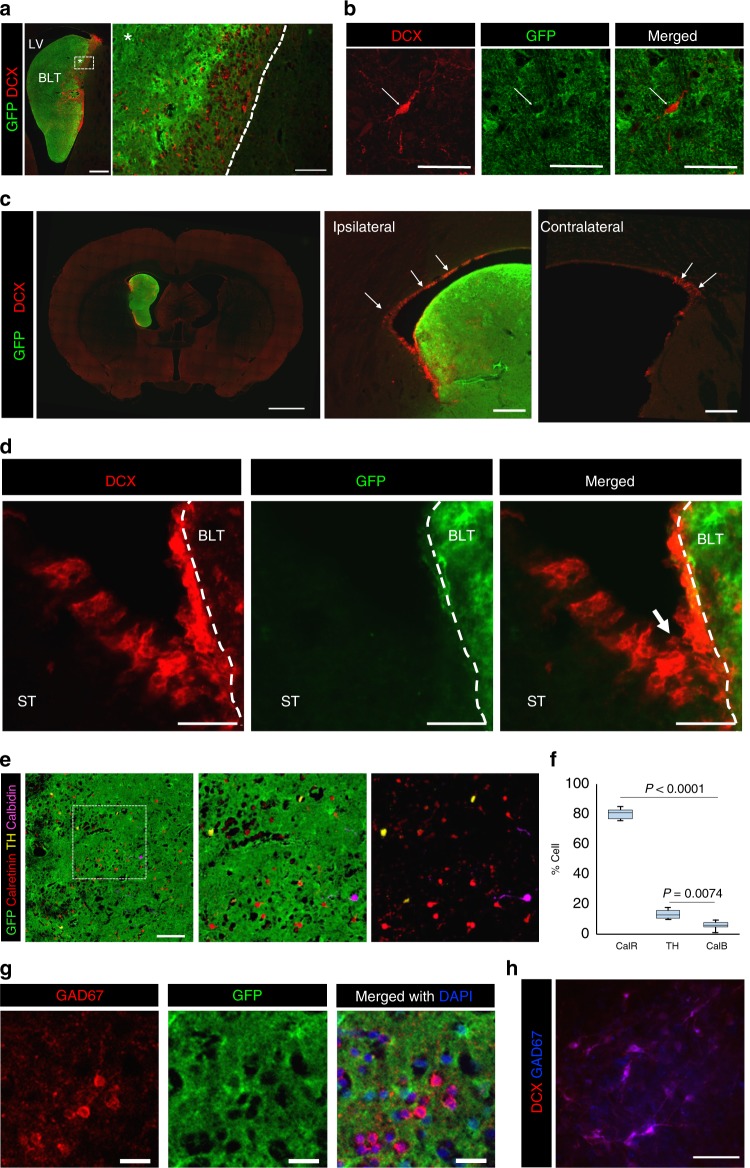
Induction of long-distance migration of neural progenitors from the host neurogenic niche into the new tissue. **a** Immunostaining with DCX showed presence of immature neurons with in the new brain-like tissue (BLT). Scale bar = 500 and 100 μm from left to right **b** These DCX-expressing cells (arrow) are negative for GFP indicating that they are of the host origin. Scale bar = 50 μm. **c** The new neurons appeared to have migrated from the adjacent ventricle wall (marked with arrows in the right two panels). Scale bars = 2 mm, 250 μm, and 250 μm from left to right. **d** High magnification image of host DCX-expressing cells migrated into the brain-like tissue (BLT) which attached into the host striatum (ST). Scale bar = 100 μm. **e** Three major subsets of SVZ-born interneurons were found within the new tissue. Scale bar = 100 μm. **f** Calretinin-expressing neurons are predominant (>80 %), TH-and calbindin-expressing interneurons (14% and 6%, respectively) were also present in the new tissue. **g** Presence of host-derived (GFP-negative) GAD67-expressing cells (GABAergic) in the new tissue. Scale bar = 25 μm. **h** DCX-expressing immature neurons also co-expressed GAD67. Scale bar = 50 μm

The origin of the host-derived neurons within the new tissue was further investigated. Because the close proximity of some of the new tissue to the host SVZ, the region where new neurons are continuously produced, it is possible these neurons could have been recruited from this niche and then migrated into the new tissue. Indeed, immunostaining with doublecortin (DCX), a marker of immature neurons, revealed a presence of GFP-negative/DCX-positive cells densely populated near the border between host and new tissue (Fig. 3a, b) indicating that they originated from the host. These DCX-positive cells appeared to migrate from the host SVZ along a wall of the lateral ventricles (Fig. 3c) and make their way into the new tissue through the interfaces of the new tissue that were in close contact or fused with the host lateral ventricle wall (Fig. 3d).

The three major SVZ-derived GABAergic interneuron phenotypes were found in the new tissue including calretinin (80%), tyrosine hydroxylase (14%), and calbidin (TH) (6%)-expressing cells (Fig. 3e, f) consistent with the hypothesis that the new neurons were of host SVZ origin. In good agreement with this finding was the presence of GFP-negative, glutamic acid decarboxylase-67 (GAD67)-expressing cells (Fig. 3g), which were also positive for DCX (Fig. 3h). The recruitment of immature neurons by the new brain graft could also be detected at 15 months post-implantation, which is well into adulthood for the animal (Supplementary Fig. 11) suggesting that the implanted cortical-derived tissue could continue to attract new neurons from the host over a long period of time, similar to what occurs in the adult olfactory bulb which continuously attracts new neurons from the SVZ in the adult rodent brain^13^. Because of evidences of such massive migration of host cells into the implant, we checked whether cells from the implant migrated into the host. Interestingly, there was no evidence of a large-scale migration from the implant into the host except for a few cells in the fimbria of hippocampus which was located in close proximity to the transplant.

To investigate whether the migration of host immature neurons was only specific to telencephalon derived-tissue, DCX-staining was performed in the tissue sections from the animals that receive implantation of midbrain-precursor cells. Interestingly, we observed that the midbrain tissue, unlike the tissue that was derived from cortical precursors, did not induce migration of neural precursors from the host SVZ (Supplementary Fig. 12). This finding is consistent with the observation that neurons found within the midbrain-derived tissue were only of the implant-origin. In contrast to the cortical-derived tissue, immunostaining with anti-GAD67 antibody showed GAD67-positive neurons in the midbrain-derived tissue that were also GFP-positive, indicating that the implanted midbrain precursors give rise to GABAergic neurons (Supplementary Fig. 13). Overall, these results show that the brain-like tissues derived from different regions of developing brain have distinct abilities to interact with the host neurogenic niche.

### Local and long-range synaptic connectivity with the host brain

**Fig. 4:**
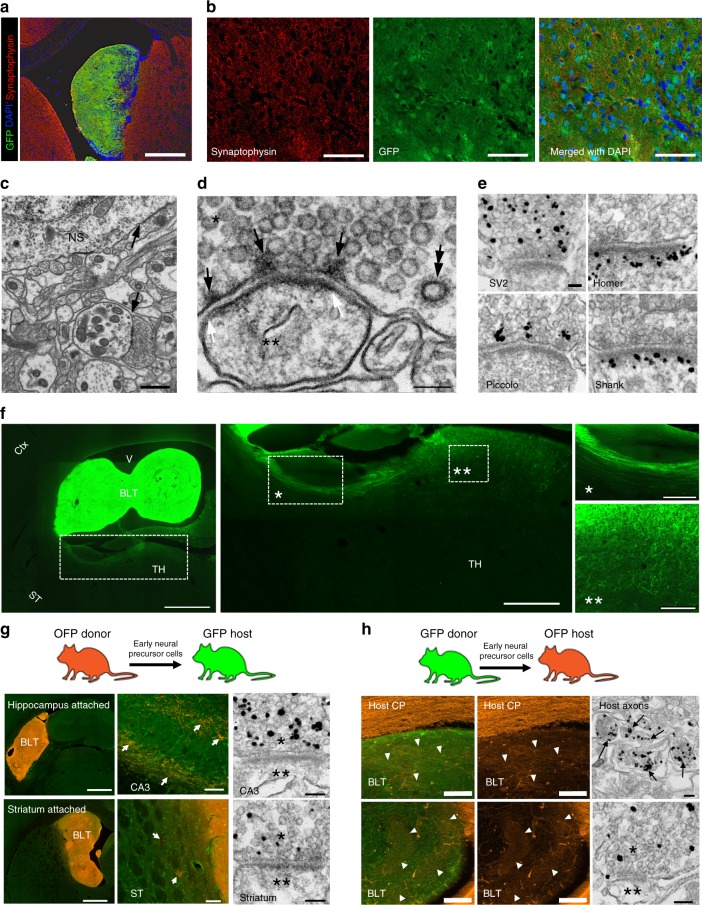
Synaptic connectivity of the brain-like new tissue with the host brain. **a** Immunostaining for synaptophysin showed widespread synaptophysin immunoreactivities throughout the new tissue. Scale bar = 1 mm. **b** Higher magnification images showed synaptophysin puncta. Scale bar = 100 μm. **c** Examination with EM showed that the implanted cells formed brain-like tissues with differentiated neuronal somas (NS), neurites, and synapses (arrows). Scale bar = 1 μm. **d** A high-magnification image of a structurally mature synapse shows clusters of synaptic vesicles, active zone materials (arrows), a coated vesicle (double arrow) in the presynaptic terminal (marked with *), and the postsynaptic density (area between two white arrows) in the spine (marked with **). Scale bar = 0.1 μm. **e** These synapses were also labeled for other markers including SV2, Homer, Piccolo, and Shank. Scale bar = 0.1 μm. **f** Long-distance projections from brain-like tissue (BLT) in the ventricle into host thalamus (TH). Scale bar = 1 mm, 500 μm, 200 μm, and 100 μm from left to right. **g** OFP donor cells from the new tissue projected into the GFP-positive host hippocampus and striatum. Immunogold staining demonstrated presynaptic terminals of the donor origin forming synapses with the host. Scale bars = 1mm, 50 μm, and 0.1 μm (top panels, left to right) and 1 mm, 100 μm, 0.1 μm (lower panels, left to right). **h** OFP host cells projected into the GFP brain-like tissues. Immunogold staining demonstrated labeled axon bundles, as well as presynaptic terminals of host origin forming synapses with dendritic element of the donor tissue. Scale bars = 100 μm in IHC panels and 0.1 μm in EM panel

Local-distance and long-distance synaptic connectivity of the brain graft were assessed. Immunostaining against synaptophysin, a synaptic vesicle marker, revealed that the signals were abundant throughout the new tissue (Fig. 4a, b). EM characterization further unequivocally identified presence of mature synapses (Fig. 4c). These synapses displayed the characteristic clusters of synaptic vesicles in the axon terminal with dense materials at the active zone (black arrows, (Fig. 4d) and clathrin-coated vesicles (double arrows) indicating active endocytosis. A prominent postsynaptic density (the borders of which are marked by white arrows) indicates that these synapses are mature excitatory glutamatergic synapses^14^. These observations are consistent with the assessment that the bulk of the neurons of the implant origin were glutamatergic. Immunogold labeling of various synaptic proteins, including SV2 (a synaptic vesicle marker), Piccolo (a presynaptic active zone marker), and Homer and Shank (two postsynaptic density markers), further demonstrated that they contained a full complement of synaptic markers in the appropriate locations characteristic of mature synapses (Fig. 4e).

To determine whether there were long-range axonal projections from the new tissue into the host, serial tissue sections were screened for GFP-positive fibers in the host brain. We found that the new tissue consistently formed connections with the host thalamus, striatum, and hippocampus (Fig. 4f, Supplementary Fig. 14). To help distinguish host components from cells originating from the implant, we generated a transgenic Lewis rat line that ubiquitously expressed orange fluorescent protein (OFP)^15^. Either GFP or OFP line was used as the donor of transplanted cells and the other as the recipient host animal. Using this double labeling model, the origin of cell types in the new tissue can be confirmed (GFP-positive or OFP-positive) consistent with assigning GFP-negative or OFP-negative to the host origin. Immunogold labeling for OFP demonstrated presynaptic terminals originating from the transplant forming synapses in the CA3 region of the host hippocampus and striatum (Fig. 4g). Additionally, long-range neural projections from the host brain into the new tissues were also observed in the grafts that attached near the hippocampus (Fig. 4h). Further characterization with immunogold EM indeed demonstrated presynaptic terminals originating from the host forming synapses with dendritic elements of neurons of the implant. Not only did the host extend axonal projections into the new tissue, the host-derived inhibitory neurons which migrated into the tissue (as discussed above in section 3 of the Results) also received synapses from glutamatergic neurons of the new tissue (Supplementary Fig. 15). Together, these results show that the brain graft can form both local and reciprocal long-distance synapses with host neurons.

### Formation of brain-like tissue in a CSF-filled cortical cavity

**Fig. 5:**
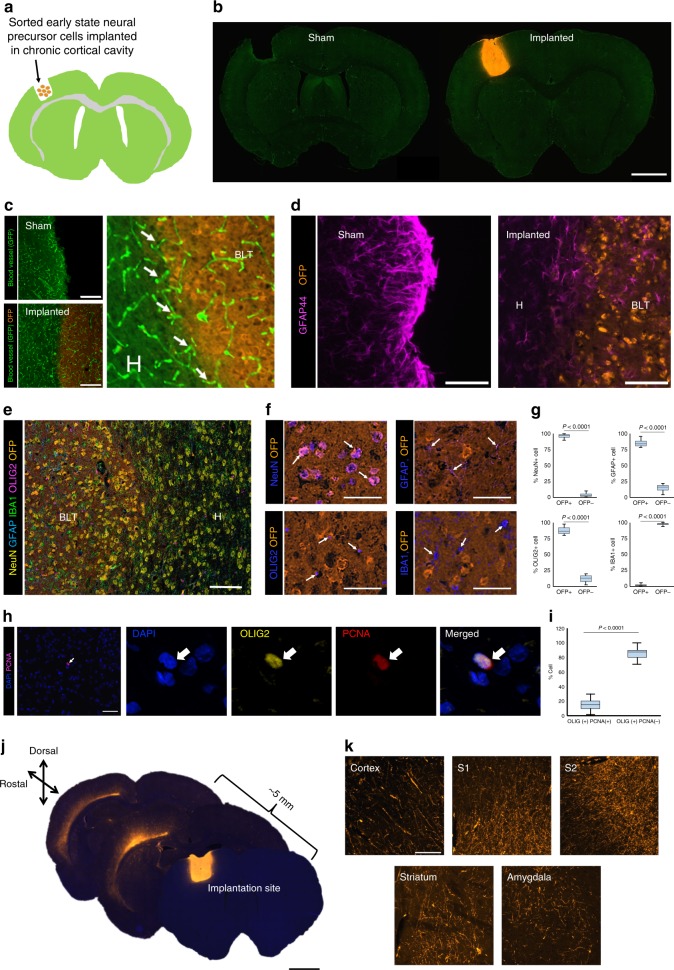
Generation of brain-like tissue within the chronic injury-induced cortical cavity. **a** Implantation of sorted OFP-positive early state neural precursor cells into the cortical cavity following chronic ablation in the adult GFP rat. **b** Brain-like tissue (orange, OFP-positive) formed within the cortical cavity by 8 weeks after implantation into the host (green, GFP-positive). Scale bar = 2 mm. **c** The brain-like tissue was vascularized by the host (GFP, highly expressed in blood vessel endothelial cells). Scale bar = 250 μm. **d** Remodeling of glia scar at the interfaces between the host and the new tissue. Scale bar = 50 μm. **e** Presence of differentiated brain cells including neurons, astrocytes, oligodendrocytes, and microglia within the new tissue. The area at the interface (boxed) was noted with seamless fusion of the new tissue into the neighboring host cortex without sign of astroglial or microglial scars. Scale bar = 100 μm. **f** Origin of neurons (NeuN+), astrocytes (GFAP+), oligiodendrocytes (Oig2+), and microglia (IBA1+) within the new tissue filled in the ablated cortex. Scale bars = 50 μm. **g** Quantification of the origin of each major cell type. **h** Immunostaining for PCNA shows a rare proliferating cell in the new cortical tissue which is also Olig2-positive identifying presence of immature oligodendrocyte precursors. **i** The oligodendrocyte precursor cells are accounted for approximately 10% of total Olig2-positive cell population. **j** The brain-like tissue formed in cortices displayed very long-distance projections (greater than 1 cm) throughout the ipsilateral hemisphere. Scale bar = 2 mm. **k** The innervations projected into several areas including cortex (S1 and S2), striatum and amygdala. Scale bars = 100 μm

Due to the propensity of the early stage neural precursors to form tissue in the CSF-rich space, it was explored whether the brain-like tissue could develop within a fluid-filled cavity that formed after chronic cortical injury. Specifically, we tested whether the permanent glial scar surrounding the injury site could be overcome and allow for an integration of the new tissue into the host cortex. In this experiment, 3-week old rats underwent mechanical cortical ablation within the forepaw area of sensorimotor cortex (approximately 5 mm^3^ lesion) in one hemisphere (Fig. 5a). Three weeks following ablation, sorted early telencephalon precursor cells were directly implanted into the cavity. In all animals, it was observed that these cells proliferated and expanded to form tissue structures that filled the entire lesion 8 weeks after implantation (Fig. 5b, Supplementary Fig. 16). The newly-formed tissue appeared to have host-derived blood vessels with similar density and morphology compared to the host vasculature (Fig. 5c). Notably, neighboring host capillaries could be seen extending into the new graft (noted by arrows in Fig. 5c).

The formation of new tissue also led to a remodeling of the glial scar around the injury site. The GFAP-positive layer which was prominent at the border of ablated tissue in sham controls was markedly reduced at the interface between the host cortex and new implant-derived tissue (Fig. 5d, Supplementary Fig. 17) similar to remodeling of ventricular wall as in the case of intraventricular implant. Immunostaining for NeuN, GFAP, Olig2, and Iba1 showed the presence of neurons, astrocytes, oligodendrocytes, and microglia within the new tissue (Fig. 5e–g). Further analysis showed that the percentages of neurons and astrocytes that were derived from the implanted precursor cells were comparable to those found in the tissue formed within the ventricle (Table 1) (For neuron; *P* = 0.4093, unpaired *t*-test, *t* = 0.8868, For astrocyte: *P* = 0.6754, unpaired *t*-test, *t* = 0.4399). In both cases, the implanted cells differentiated into neurons more than astrocytes at approximately 1.5–1.8 to 1 ratio. Interestingly, we observed two key differences between the new tissue formed in the cortical cavity and the ventricle. First was the absence of the migration of host inhibitory neurons into the cortical implant (reflected in Fig. 5g), presumably due to its location that was not connected to the host neurogenic niche. Another difference was that oligodendrocytes in the cortical-cavity implant were derived mostly from the implanted cells (Fig. 5g), unlike those found in the intraventricular implant, which were mostly derived from the host suggesting the microenvironment could affect the oligodendrocyte recruitment.

**Table 1:** The average numbers of neurons (NeuN+) and astrocytes (GFAP+) in the tissues that formed in ventricles and cortical ablated cavity

Cell type	Number of cells in the implant*	
	Ventricular implant	Cortical cavity implant
Neuron	41.3 ± 7.3	37.0 ± 6.6
Astrocyte	21.8 ± 4.6	23.3 ± 5.0

*Number of cells per 200 μm × 200 μm ROI. Data represents means ± standard deviations

Consistent with the intraventricular brain-like tissue, very little proliferative activity was detected at 8 weeks post-implantation of neural precursors into the cortical ablation site and all could be attributed to few proliferating Olig2(+) oligodendrocyte precursors (Fig. 5h, i). Strikingly, there was a remarkable long-distance outgrowth from the tissue that extended approximately 1 to 2 centimeters throughout the ipsilateral hemisphere (Fig. 5j) and projecting into all cortical layers, somatosensory areas, striatum, and amygdala (Fig. 5k). While the outgrowth from the new tissue into the host brain was prominent, there was also evidence of the extension of GAD67-positive processes from the host into the new tissue (Supplementary Fig. 18), which is likely to compensate for the absence of host inhibitory neurons. The results show that an integrated complex neural tissue can form in a CSF-filled, injured area of the cortex that leads to remodeling of the astroglial scars and generates long-range connectivity to the host brain.

## Discussion

There has been extensive work on transplanting neural precursor cells in the brain. Most studies have focused on transplanting cells into specific sites within brain tissue and have shown that cell survival declines substantially within a few weeks after transplantation^16–18^. To a much lesser extent, injection of cells into CSF for whole-brain cell delivery has demonstrated that injected cells either disseminate throughout the brain or integrate into discrete regions without forming a complex tissue structure^19,20^. In this study, early stage precursor cells from embryonic cortical or midbrain region survived and expanded robustly in the CSF of young adult rodents and fully differentiated into normal cell phenotypes that gave rise to a brain-like tissue that seamlessly integrated into the host brain via a rearrangement of the host ependymal layer. The integration of the new tissue into the host brain was also supported by intact blood vessels from the host. This present study also highlighted the host ability to supply appropriate cells types including microglia, oligodendrocytes, and inhibitory neurons to the new tissue. Overall, the results demonstrated that the CSF could be used as in vivo niche for implanted early-stage neural precursor cells to develop into brain-like tissues that are capable of forming mature synaptic connectivity and extensive long-range axonal projections with the host brain.

The absence of proliferative cells originating from the graft and no apparent increase in tissue size beyond 8 weeks after the implantation indicated that the new tissue was not malignant. Nor did the newly-formed tissue have characteristics of benign glioneuronal tumors. No evidence of bi-nuclear neurons, astrocytes with mitotic activity, and long-term growth suggest the structures are more similar to normal tissue than typical glioneuronal tumors^12,21^. In most glioneuronal tumors, the percentage of proliferation is typically in the range of 1–2%. However, within the graft, the numbers of proliferating cells were well below 1%. For the case of cortical-derived tissue, proliferating cells were of the host origin which attributed to the oligodendrocytes precursors which are known to divide slowly in the adult brain^22,23^. The lack of apparent growth over a prolonged period of time (up to 15-month post-implantation) and the absence of distress or neurological symptoms provide further support that the structures have mature brain-like tissue phenotypes.

Another interesting aspect of the present study was the evidence of an interaction of the graft with the host SVZ, which induced long-distance migration of GABAergic neurons into the new tissue. Although the exact molecular mechanism of this process is unclear, it is known that GABAergic precursors migrate long distances from the ganglionic eminence (GE) during early development into the developing neocortex where they are functionally integrated^24,25^. Thus, it seems probable that the migration observed here was orchestrated by chemotactic signals generated by the developing tissue. Interestingly, the midbrain precursor-derived tissue did not recruit GABAergic neurons from the host SVZ, unlike in the case of cortical precursor-derived tissue. Possible explanation for this is the midbrain region has its own source of GABAergic neurons that originate within the dissected mesencephalic region^26^ which could lead to inhibition of migration of SVZ-born neurons from the host.

There has been much interest in using transplanted neural cell precursors to extend axons especially in injured spinal cord. Recently, Lu and colleagues reported that grafted rat spinal-cord-derived neural stem cells and human-derived neural precursors transplanted into the severed spinal cord of adult rats using a fibrin scaffold and growth factor cocktails could survive and extend axons from the injured spinal cord into the host^27,28^. Until now, it was not clear whether the long-range axonal projections could be achieved in other models beyond spinal cord transplantation. Our findings demonstrated such remarkable ability of multipotent neural stem cells to extensively extend long distance axonal projections into the host brain and that connectivity between the host brain and new graft could occur even though the tissue graft was located outside of the brain’s parenchyma. These results indicate the brain has remarkable plasticity to integrate a new tissue and add new long-distance connections even at an age beyond developmental critical periods.

Stem cell-based approaches hold great promise for reconstruction and replacement of brain tissue that is lost to injury or neurodegenerative diseases. However, cell transplantation studies to date, have shown limited evidence of success in rebuilding a complex brain tissue following injury or ischemic stroke^29–31^. Based on our observation that brain-like tissue that formed within the ventricles could integrate and extend neural processes into the host, it may be possible to use early stage precursor cells to reconstruct brain tissue that is lost after brain injury. Commonly, the injured area such as in ischemic stroke or cortical impact, transforms into a cavity that is filled with CSF fluid and cellular debris and surrounded with glial scar. Therefore, we tested whether the sorted early neural precursors could survive in such environment and develop into brain-like tissue in the cavity following the chronic cortical ablation. In this experiment, it was found that the precursor cells survived, proliferated, and developed into new tissue in a CSF-filled cavity within the adult rat brains. Furthermore, the newly formed tissue in the ablated cavity was capable of remodeling the glial scars bordering the injury site, which led to integration and formation of long-distance connectivity with the host brain. The results show intrinsic property of the early stage neural precursor cells to robustly expand and develop into brain-like tissue in both brain ventricular space and cavity. The important questions remain what the phenotype of these early neural stem/precursor cells is and whether late neural progenitors have the same potential. Another good question to be addressed in future studies is whether these cells have the capability to organize into the layered cytoarchitectural structure similar to the host cortex with layer-specific neuronal phenotypes and, if so, whether these form functional innervation circuits with their appropriate targets.

Taken together, our results warrant future work that will aim to explore whether the integrated brain tissue structures could be formed using isogenic early stage precursor cells that are derived from induced pluripotent stem cells, which could pave the way toward brain tissue repair through autologous implantation using patient-derived cells. It may be possible to implant more sophisticated neural tissue, such as in vitro-grown neural organoids^32,33^, to further derive specific brain structures (e.g., cortices with laminar pattern). Importantly, based on the observation of long-range connectivity and mature synapse formation, the ability of this brain-like tissue to affect brain function and behavior should be explored.

## Methods

### Animal procedures

All animal procedures were handled according to the Institute of Laboratory Research guidelines and were approved by the Animal Care and Use Committee (ACUC) of the National Institute of Neurological Disorders and Stroke.

### Preparation of neural stem cells

E14 green fluorescent protein (GFP) embryos from Lewis rats were isolated according approved ACUC protocol and dorsal telencephalic region of developing cortical tissues were carefully dissected under magnifying scope. The tissues were dissociated using a papain-based enzyme solution. These cells were washed and maintained in neurobasal media prior to sorting. To purify the early neural stem/precursor cells, fluorescence activated cell sorting (FACS) was used to remove lineage-committed neuronal and glial progenitors and their post-mitotic counterparts, as well as to deplete non-neural cell phenotypes (microglia, endothelial cells, pericytes) from the uncommitted lineage-negative (Lin-) neural stem/precursor population using a previously published method^8^. Briefly, telencephalic cell dissociates were stained using a panel of antibodies targeting the following surface markers: CD11b (to identify microglia), CD31 (to identify endothelial cells), NG2 proteoglycan (to identify pericytes), a cocktail of antibodies targeting A2B5, CD15 and CD24 (to identify neuroglial progenitor cells), GLAST (to identify radial glial cells and differentiating astrocytes), O4 (to identify oligodendroglial progenitors and their differentiating progeny), and a cocktail of antibodies targeting CD57, PSA-NCAM and GT1b gangliosides, via binding of tetanus toxin C-fragment (TnTx) and anti-TnTx antibody, to identify neuronal progenitors and differentiating neurons. Lin(-) cortical NSCs were then purified by applying a lineage-dumping sorting protocol excluding all cell phenotypes expressing any of the markers listed above. Finally, a uniform single cell suspension of 5 × 10^4^ Lin(−) cells per μL was prepared in neurobasal media (ThermoFisher Scientific, MA) supplemented with growth factors (bFGF and EGF, 20 ng per mL) and kept at 4 °C prior to the implantation.

### Cell implantation

Twenty one-days-old to 24-days-old Lewis rats (40–60 g body weights) were used as recipients. The animals were anesthetized under 5% isofurane in a 30% oxygen/70% nitrogen (oxygen-enhanced air) gas mixture. After anesthesia was induced, isoflurane level was adjusted to 2%. Under sterile conditions, a 1 mm burr hole was drilled in the skulls of the animals (+1.5–1.6 AP and +1.5–1.6 ML from bregma) above the lateral ventricles using stereotaxic coordinates from the Paxinos & Watson rat brain atlas^34^. Cell suspension was loaded into a 10 μL glass syringe (Hamilton, MA) equipped with a 31-gauge needle. The needle was levered slowly and placed at a 4-mm depth from skull surface. Five microliter of cell suspension was slowly levered using a hand push over a 1-min period. After injection, the needle was left in situ for 3 min before removing. The burr hole was sealed with bone wax and the skin was sutured. Immediately after surgery, the animals were given analgesic (ketoprofen, 5 mg per kg). The animals were monitored for any sign of complication and returned to their home cages. Body weights were measured weekly.

### Magnetic resonance imaging of the new tissue formation

MRI was performed following implantation to monitor growth kinetics of the new tissue in the ventricle. All MRI experiments were done on an 11.7 T animal MRI system (30 cm 11.7 T horizontal magnet, Magnex Scientific, Oxford, England; MRI Electronics, Bruker Biospin, Billerica, MA) with a 12-cm integrated gradient shim system (Resonance Research Inc, Billerica, MA) using a custom-built volume transmit coil and a custom built, receive-only 2-coil array surface coil. Flash 3D gradient echo sequences were used for all MRI acquisitions. For in vivo imaging, the following parameters were used: field of view (FOV) = 1.92 cm^3^, matrix size 256 × 256 × 256 (100 μm isotropic resolution), 12.5 kHz bandwidth, TE = 8 ms, TR = 25 ms, and flip angle = 8°.

### BBB permeability assessment

For MRI assessment of BBB permeability, rats at 16 weeks post-implantation were infused with GdDTPA (200 mol per kg) via tail vein injection. MRI was performed prior to the infusion of GdDTPA and serial MRI was then performed immediately after the infusion and every 20 min for 2 h. For the Evans blue assay, rats at 16 weeks post-implantation were infused with Evans blue dye (EBD) solution through a tail-vein (2% in PBS, 2 mL per kg) under anesthesia with 2% isofurane. 20 min after the infusion, the rats were euthanized, and the brains were removed and processed for histological assessment of EBD accumulation.

### Cell implantation into chronic cortical cavity

Twenty one-days-old to 24-days-old Lewis GFP transgenic rats were used as recipients. The animals were anesthetized under 5% isofurane in a 30% oxygen/70% nitrogen (oxygen-enhanced air) gas mixture. After anesthesia was induced, isoflurane level was adjusted to 2%. Under sterile conditions, a 2-mm burr hole was drilled in the skulls of the animals (+1.5–1.6 AP and +1.5–1.6 ML from bregma) using stereotaxic coordinates from the Paxinos & Watson rat brain atlas. The 1.5 mm × 1.5 mm × 2.0 mm ablated area was created by placing the drilling tip into the cortex for 5 s. After bleeding stopped, the burr hole was sealed with bone wax and the skin was sutured. Immediately after surgery, the animals were given analgesic (ketoprofen, 5 mg per kg). The animals were monitored for any sign of complication and returned to their home cages. Three weeks following ablation, approximately 2 × 10^4^ sorted telencephalic precursor cells in 1 μL were injected into the ablated cavity (*n* = 3) in similar procedures as described above, except that the needle was levered to 1.5 mm to minimize injection of cells into the host brain tissue. Sham animals (*n* = 2) received only media (supplemented with EGF and bFGF).

### Immunohistochemistry for characterization of cell phenotypes

10-μm thick brain coronal or sagittal sections were immunoreacted for 1 h at room temperature (RT) using 1 μg per mL final concentration (diluted in PBS supplemented with 1% bovine serum albumin, PBS/BSA) of the following primary antibodies (vendor source and product number are indicated in parentheses): mouse IgG1 anti-rat endothelial cell antigen (RECA1) (Abcam, ab9774), mouse IgG1 anti-CD68/ED1 (Thermo Fisher Scientific, MA5-16654), mouse IgG1-calretinin (EMD Millipore, MAB1568), mouse IgG2a anti-Calbindin (Abcam, ab75524), mouse IgG2a Olig2 (EMD Millipore, MABN50), mouse IgG2b anti-proliferation cell nuclear antigen (PCNA) (Abcam, ab184660), mouse IgG3 anti-glutamic acid decarboxylase 67 (GAD67) (Santa Cruz Biotechnology, sc-28376), mouse IgG2a anti-S100 (EMD Millipore, MAB079-1), mouse IgM anti-synaptophysin (EMD Millipore, MAB329), rat IgG2a anti-myelin basic protein (MBP) (EMD Millipore, MAB386), chicken IgY anti-glial fibrillary acidic protein (GFAP) (EMD Millipore, AB5541), chicken IgY anti-Neurofilament (NF) (EMD Millipore, AB5539), chicken IgY anti-Tyrosine Hydroxylase (TH) (Abcam, ab76442), rabbit IgG anti-Iba1 (Wako Chemicals, 019-19741), rabbit IgG anti-Doublecortin (DCX) (Abcam, ab18723), rabbit IgG anti-Glutaminase (Abcam, ab113509), rabbit IgG anti-platelet derived growth factor beta (PDGFR- β(Abcam, ab32570), guinea pig IgG anti-NeuN (EMD Millipore, ABN90P). Select combinations of immunocompatible primary antibodies (i.e., antibodies from a different host or belonging to a different immunoglobulin class or subclass) from the list above were also used for multiplexing two or more biomarkers at the same time, as detailed in the results section. The sections were then washed in PBS/BSA and immunoreacted using a 1 μg per mL of the appropriate secondary antibodies (Thermo Fisher Scientific, Li-Cor Biosciences) conjugated to one of the following spectrally compatible fluorophores: Alexa Fluor 350, Alexa Fluor 405, Alexa Fluor 430, Alexa Fluor 488, Alexa Fluor 546, Alexa Fluor 594, Alexa Fluor 647, IRDye 680LT, or IRDye 800CW. Some sections were also counterstained with 1 μg per mL DAPI (Thermo Fisher Scientific) to facilitate cell counting. The slides with labeled tissue sections were then coverslipped using Immu-Mount medium (Thermo Fisher Scientific, MI). All sections were imaged using an Axio Imager Z.2 multi-channel scanning fluorescence microscope (Carl Zeiss, Thornwood, NY) equipped with a 20X Plan-Apochromat (Phase-2) objective (Carl Zeiss), a high resolution ORCA-Flash 4.0 sCMOS digital camera (Hamamatsu Photonics, Japan) sensitive to a broad-spectrum of emission wavelengths, including those approaching infrared, a 200W X-Cite 200DC broad-spectrum light excitation source (Lumen Dynamics), and 10 self-contained excitation/dichroic/emission filter sets (Semrock, Rochester, NY) optimized to detect the following fluorophores with minimal spectral crosstalk: DAPI, Alexa Fluor 350, Alexa Fluor 405, Alexa Fluor 430, Alexa Fluor 488, Alexa Fluor 546, Alexa Fluor 594, Alexa Fluor 647, IRDye 680LT, and IRDye 800CW. Each labeling reaction was sequentially captured using filtered light through an appropriate fluorescence filter set and the images individually digitized at 16-bit resolution using the ZEN imaging program (Carl Zeiss). An appropriate color table was applied to each image to either match its emission spectrum or to set a distinguishing color balance. The pseudocolored images were then converted into TIFF files, exported to Adobe Photoshop and overlaid as individual layers to create multi-colored merged composites.

### Immunohistochemistry for assessment of long-range synapses

Thirty-micrometer thick brain coronal sections immunostained with anti-GFP and anti-OFP antibodies using a standard procedure for free-floating immunohistochemistry. Primary antibodies used for staining were mouse IgG1 monoclonal anti-mCherry (cross reacts with OFP) (Clonetech, 632543) and chicken polyclonal anti-GFP (Abcam, ab13970). These were visualized using Alexa Fluor 594 conjugated goat anti-mouse IgG1 and Alexa Fluor 488-conjugated goat anti-chicken IgY secondary antibodies (Thermo Fisher Scientific), respectively. Sections were mounted onto slides and coverslipped using Immu-Mount and images with a Nikon Eclipse Ti microscope (Nikon, CA) using a ×20 objective.

### Ultrastructural analysis

Rats were deeply anesthetized with isoflurane, and intracardially perfusion fixed with 2% glutaraldehyde + 2% paraformaldehyde (EMS, Fort Washington, PA) in 0.1 N sodium cacodylate buffer at pH 7.4. Perfusion pressure was maintained at 130 mm Hg with a Masterflex L/S Economy Pump System (Cole-Parmer, Vernon Hills, IL). The fixed brain was dissected and immersed in the same fixative until ready for further vibratoming into 100 µm coronal sections. The brain sections containing the attached transplants in the ventricle were further fixed and stored in 2% glutaraldehyde in 0.1 N cacodylate buffer. Slices were then washed in buffer, treated with 1% osmium tetroxide in 0. N cacodylate buffer at pH 7.4 for 1 h on ice, then block stained with 0.25% uranyl acetate in acetate buffer at pH 5.0 overnight at 4 °C, dehydrated in graded ethanol solutions, and embedded in epoxy resin. Thin sections were counter stained with uranyl acetate and lead citrate and examined with a JEOL 1200 EXII transmission electron microscope. Images were photographed with a bottom-mounted digital CCD camera (AMT XR-100, Danvers, MA, USA).

### Immunogold labeling

Rats were perfused according the protocol described above but using 4% paraformaldehyde in PBS as fixative. In order to avoid over-fixation, total fixation time was about 60 min based on the beginning of flow of the fixative into the heart until the time the brain was vibratomed into 100 µm thick coronal sections. The brain sections were stored in PBS at 4 °C for no more than a week before they were immuno-labeled, free-floating in 24-well cell culture plates. All steps were carried out at room temperature unless otherwise indicated. Samples were made permeable and blocked with 0.1% saponin and 5% normal goat serum in PBS for 40–60 min, incubated with primary and then secondary antibodies (Nanogold, at 1:200, Nanoprobes, Yaphand, NY) for 1 h, fixed with 2% glutaraldehyde in PBS for 30 min, and stored at 4 °C in fixative. Samples were then washed thoroughly in deionized water, silver enhanced (HQ kit, Nanoprobes), treated with 0.2% osmium tetroxide in 0.1M phosphate buffer at pH 7.4 for 30 min on ice, then block stained with 0.25% uranyl acetate in acetate buffer at pH 5.0 for 1 h at 4 °C, dehydrated in graded ethanol, and embedded in epoxy resin. Antibodies used for immunogold staining were as follows: mouse monoclonal antibody (mouse mAb) against SV2 (1:500, clone 10H3), was a gift from Dr. Erik S. Schweitzer (University of California Los Angeles, CA, USA); rabbit polyclonal antibody (rabbit pAb) against synaptophysin (1:250) was from DAKO (Glostrup, Denmark); guinea pig polyclonal antibody against Piccolo (1:100), which was a gift from Dr. Eckart Gundelfinger (Leibniz Institute for Neurobiology, Magdeburg, Germany). Finally, mouse monoclonal antibody against Homer 1 (pan Homer 1, clone 2G8), rabbit polyclonal antibodies Homer 1b/c (raised against aa 152–354 of human Homer 1b), and Shank3 were from Synaptic Systems (Göttingen, Germany)

### Generation of orange fluorescent protein (OFP) rat

Transgenic animals were produced using techniques that have been described^35^. Five to six weeks old Lewis females weighing over 150 g were injected intraperitoneally with 40 µg LHRH (Sigma-Aldrich, L4513), followed 48 h later with 20 IU per head pregnant mare serum (PMS, National Hormone and Peptide Program) and then followed at 96 h with 30 IU human chorionic gonadotropin (hCG, Pregnyl, Merck, Sharp and Dohme). After the last of the hormone injection, the females were mated with singly housed Lewis males. The zygotes were harvested in the next morning. A DNA plasmid containing the CAG promoter (CMV enhancer and chicken beta actin promoter) and the mOrange fluorescent (OFP) protein gene^15^ was purified using endotoxin free reagents and the fragment containing the transgene was subsequently purified from an agarose gel. Pronuclei of zygotes were injected with DNA at 5 ng per µl concentration. Recipient Sprague-Dawley females were prepared in parallel with donor animals by injecting with LHRH 80 µg per head and mating with vasectomize males. Embryos were transferred to the oviducts of females that had mated. Pups were born and screened for expression of the transgene in their skin. These animals are now available from the Rat Resource and Research Center (catalog# 746 LEW-Tg(CAG-OFP)Pic).

### Statistical analysis

Data are expressed as means ± standard deviation. Statistical analyses were performed using GraphPad (La Jolla, CA, USA). Comparison between the two groups, when possible, were made using Student’s unpaired *t*-test. Significance was determined at *P* < 0.05.

### Data availability

The datasets generated during and/or analyzed during the current study are available from the corresponding author on reasonable request.

## Electronic supplementary material

Supplementary Information

Description of additional supplementary items

Supplementary Movie 1

Supplementary Movie 2
