# Supplementary material for: Neural precursor cells form integrated brain-like tissue when implanted into rat cerebrospinal fluid
Source: Commun Biol. 2018 Aug 14;1:114. doi: 10.1038/s42003-018-0113-8 (PMC6123740; doi:10.1038/s42003-018-0113-8)
Supplement: Supplementary file 1 — Supplementary Information [file 42003_2018_113_MOESM1_ESM.pdf]

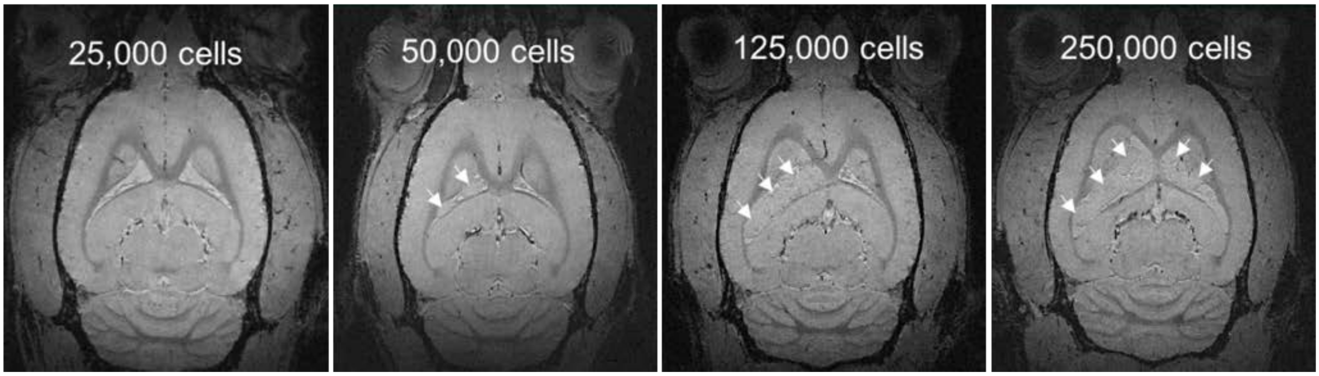

Supplementary Figure 1. Cell dose experiment. Minimum numbers of cells that are required to form brain-like tissue in the CSF was found to be at least 50,000 cells.

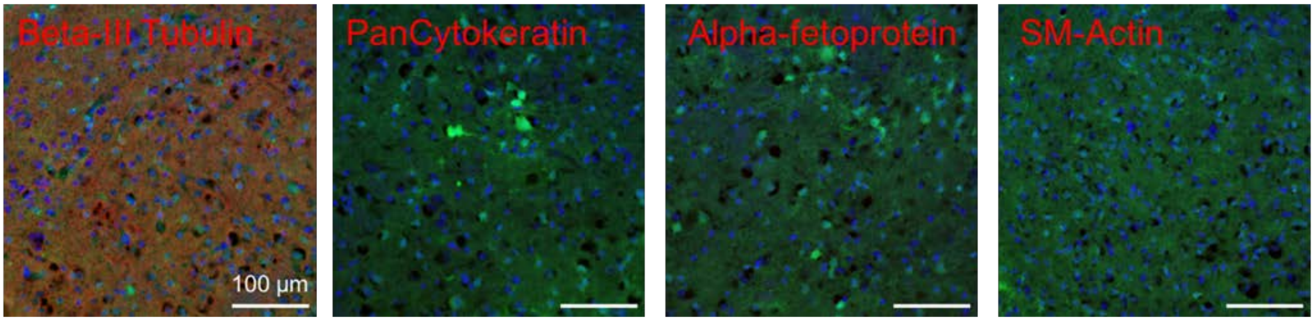

Supplementary Figure 2. New tissues were not teratomas. Immunostaining for Beta-III tubulin showed that brain-like tissue maintained phenotype of neuralectodermal lineage and lacked characteristics of teratoma including absence of cartilage structure, and was negative for PanCytokeratin, Alpha-fetoprotein, SM-actin markers.

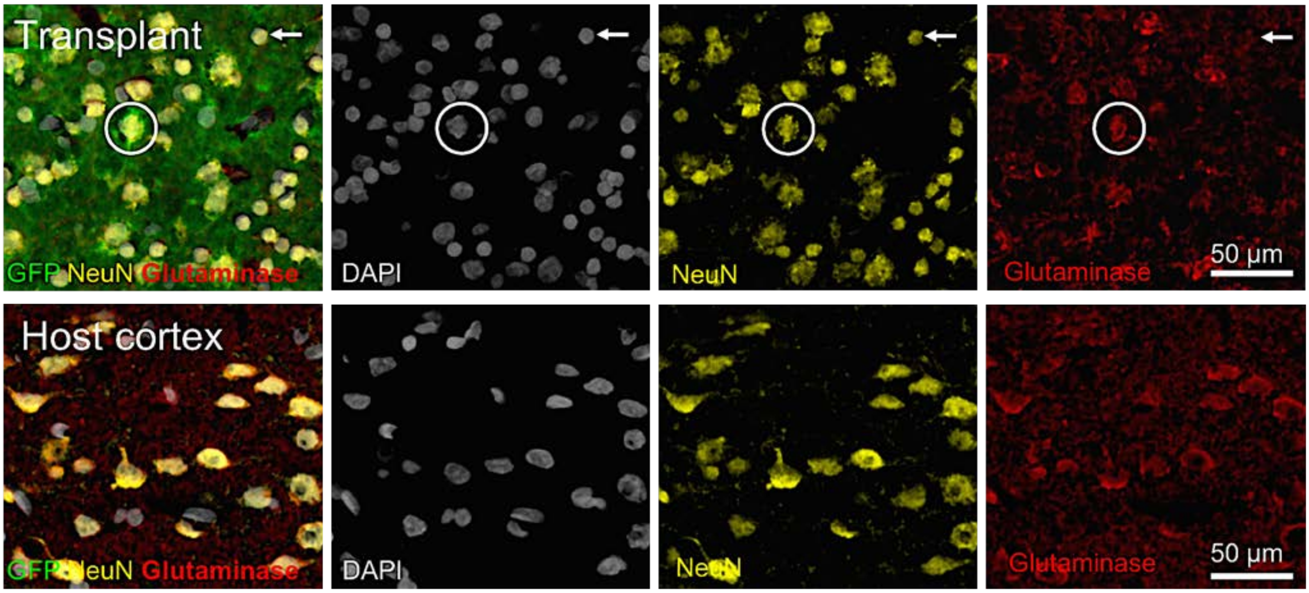

Supplementary Figure 3. Neurons of the new tissues were glutamatergic neurons. Immunostaining with anti-glutaminase, a marker for glutamatergic neuron. Top panels showed that neurons derived from implanted cells (GFP-positive, circled) were glutamatergic neurons whereas the host-derived neurons (arrowed) were not positive for glutaminase. Lower panels showed typical glutamatergic neurons in the host cortex.

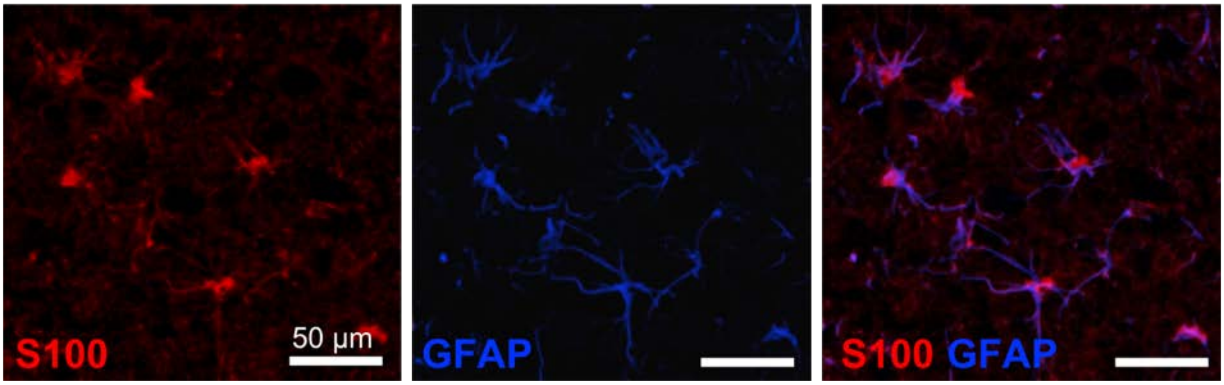

Supplementary Figure 4. Co-immunostaining with S100 (cell body) and GFAP (cell processes) showed that implanted cells gave rise to mature astrocytes

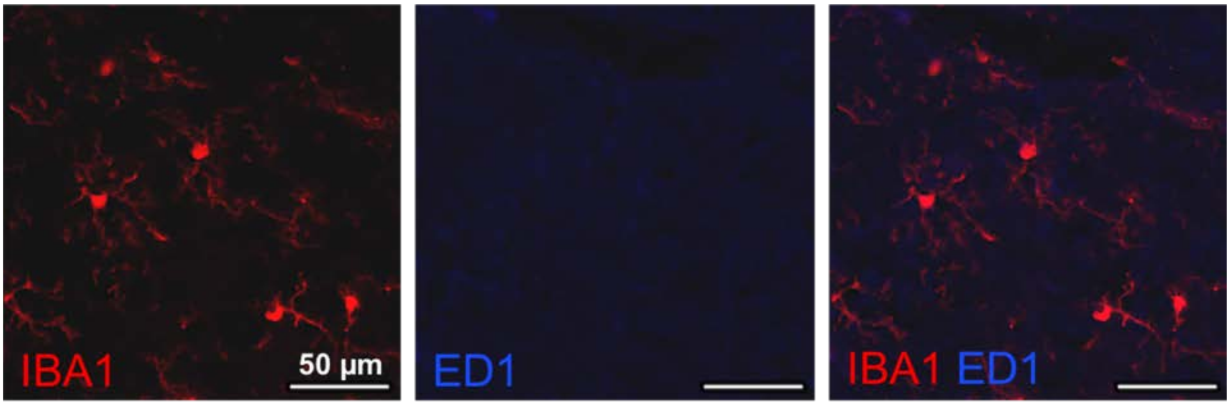

Supplementary Figure 5. Host microglia in the brain-like tissue maintained normal ramified morphology and did not express ED-1, which is a marker of activated microglia in the CNS.

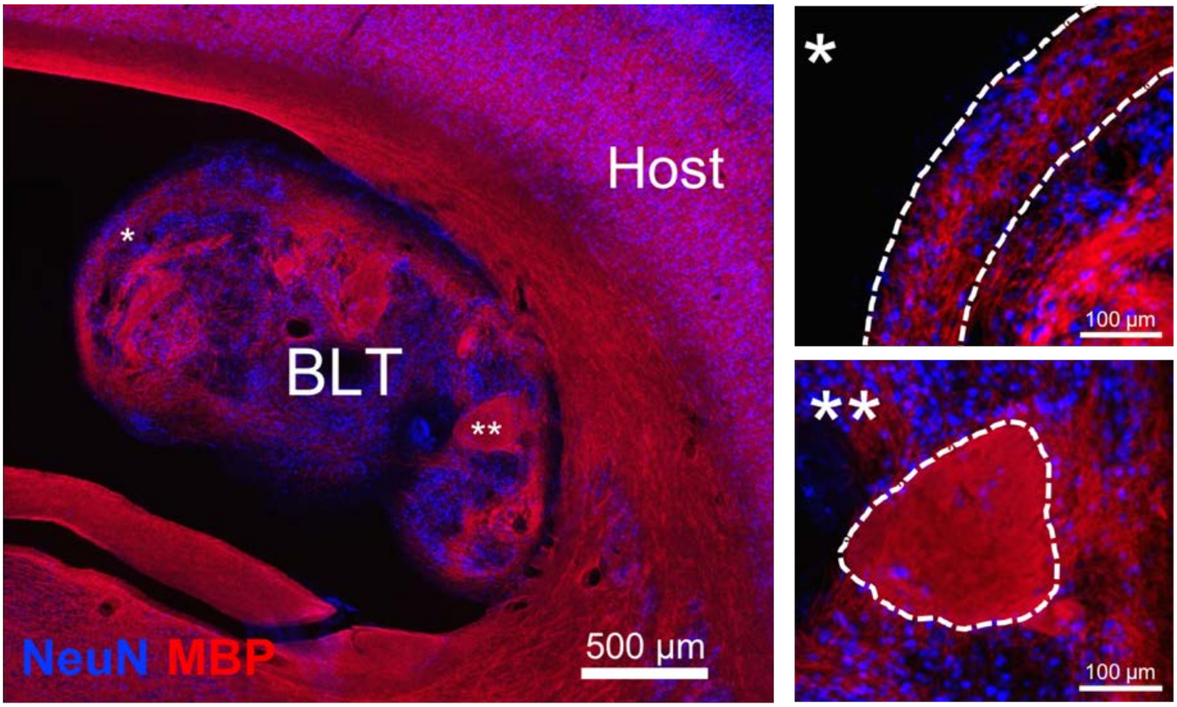

Supplementary Figure 6. New tissue exhibited widespread pattern of myelinated fibers. Immunostaining for NeuN and MBP showed large-scale structures with continuous myelination pattern that contained sparse myelination fiber that closely associated with neuronal bodies forming tract-like structure (*) and dense myelinated bundles containing few neuronal nuclei (**)

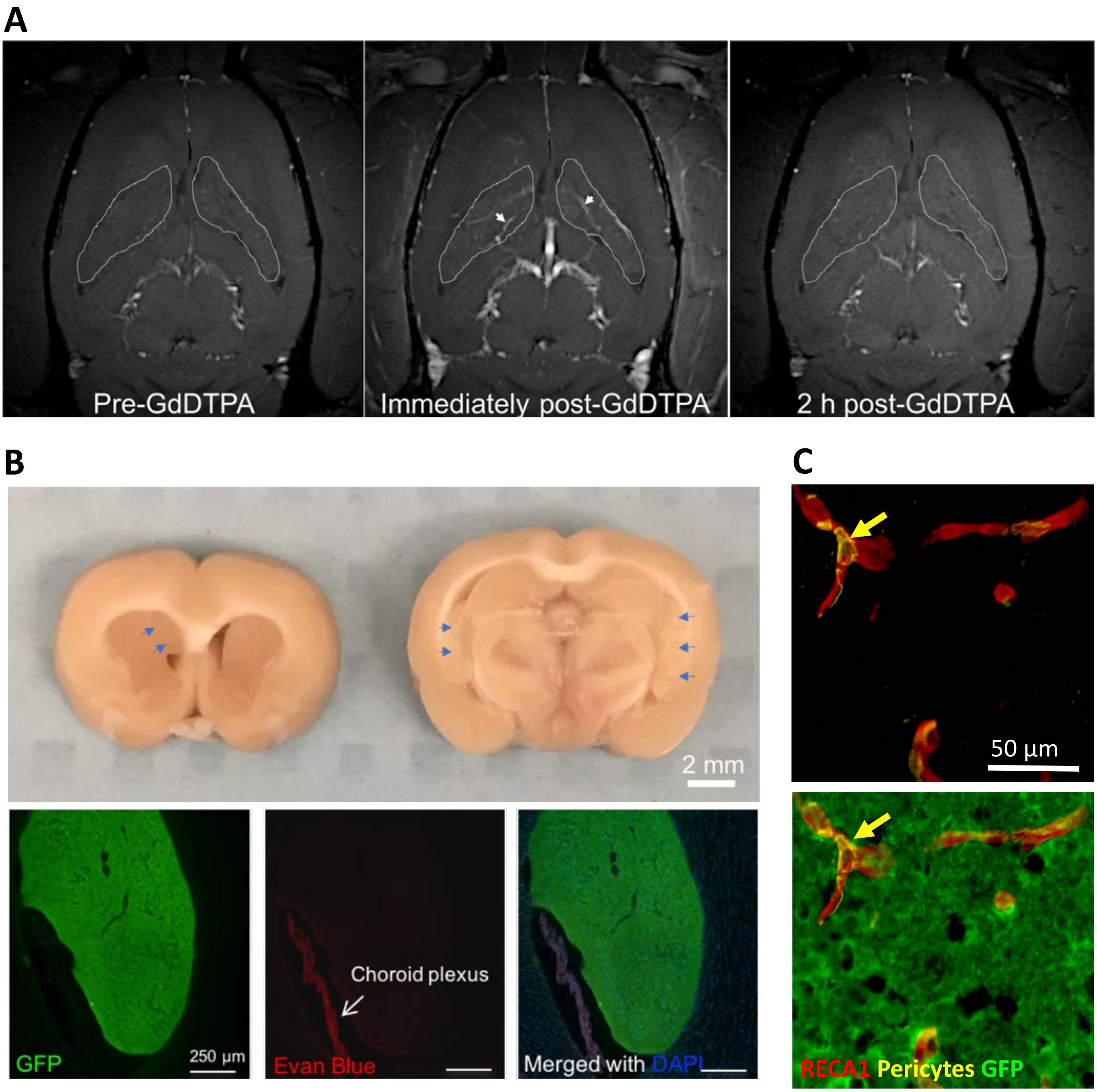

Supplementary Figure 7. Brain-like tissues were vascularized with an intact blood vessels. (A) MRI showed no detectable accumulation of the contrast agent outside in the organoid tissue (white margin). Only blood vessels (marked by white arrows) were enhanced upon administration of GdDTPA MRI contrast agent. (B) Assessment with Evan Blue dye (EBD) showed no extravasation of EBD-plasma protein complex in the new tissue (marked by arrows) or host brain tissue. The only place where EBD fluorescence signal could be detected was in choroid plexus, which lacks BBB. (C) Presence of host pericytes (GFP-negative) in the capillary branches in the brain-like tissue.

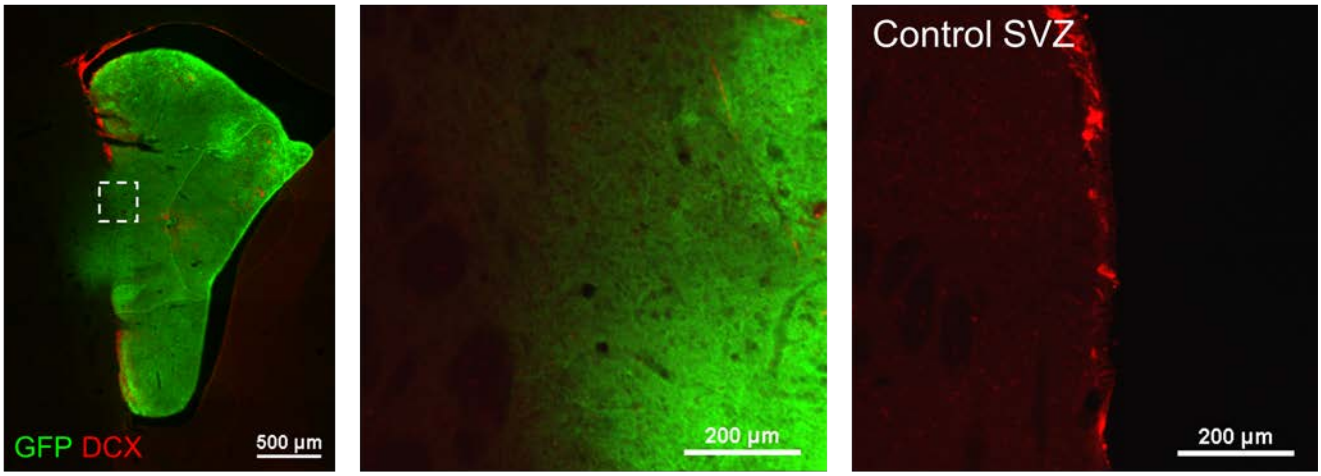

Supplementary Figure 8. Fusion of the new tissue with the host SVZ. Immunostaining for doublecortin (DCX), a marker of immature neurons, showed the integration of brain-like tissue (GFP-positive) with the host SVZ (DCX-positive). An image of DCX-labeled SVZ from a control animal is shown on right.

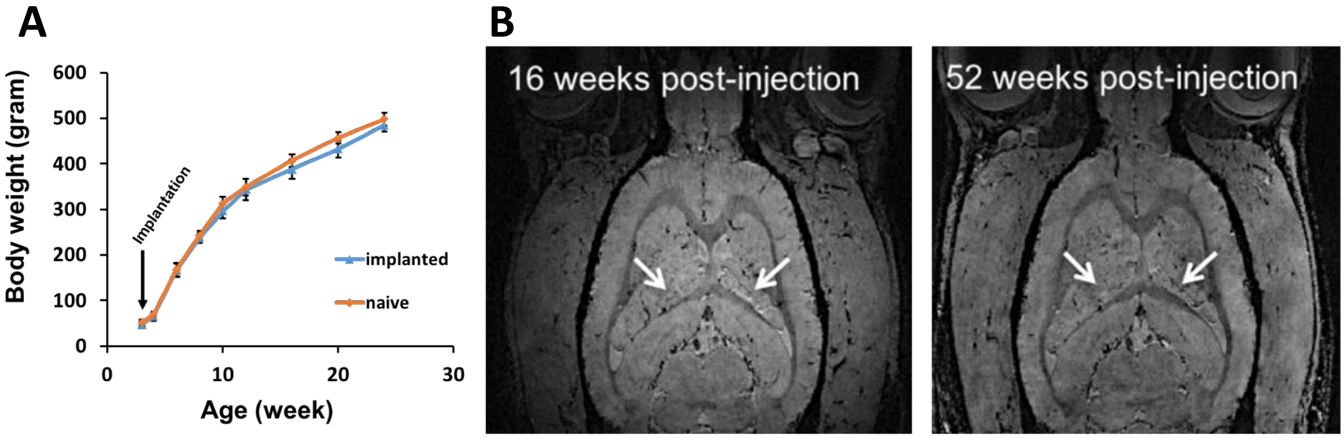

Supplementary Figure 9. Effect on intraventricular tissue on growth rate of the rats and stability the new tissue (A) Growth rate of host rats following cell implantation was comparable to that of the control rats (B) long-term stability of the brain-like tissue assessed after 12-months post-implantation showed that the new tissue remained within the ventricles

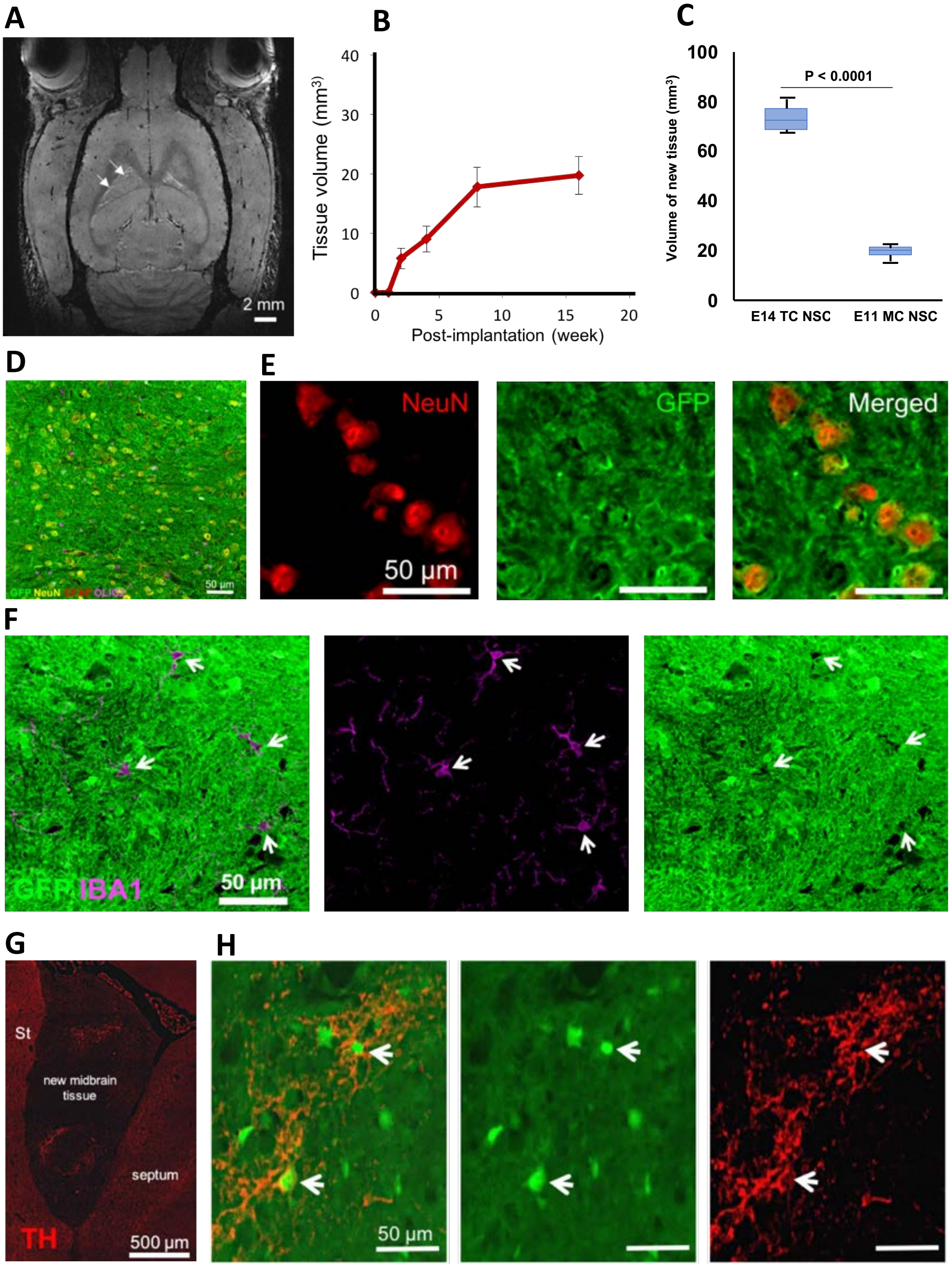

Supplementary Figure 10. Formation of brain-like tissue from midbrain precursor cells. (A) MRI image showed tissue in the rat ventricle at 8 weeks post-implantation derived from E11 mesencephalic cells. (B) MRI growth kinetic of midbrain-derived organoids in the ventricles of the rat brains. (C) Implantation of 2.5x10^5 mesencephalic precursor cells led to a tissue volume of 75% less than the same number of telencephalic precursor cells (n = 4, $p < 0.0001$, unpaired t-test, $t = 0.7461$). (D) Immunostaining for NeuN (neuron), GFAP (astrocyte), and Olig2 (oligodendrocyte) showed presences of neurons, astrocytes and oligodendrocytes in midbrain-derived tissue. (E) Neurons within midbrain-derived tissue were all GFP-positive indicating that these cells were of the implant origin. (F) Immunostaining with IBA1 confirmed a presence of microglia in the tissue and that there were derived from the host (GFP-negative). (G) Tyrosine hydroxylase immunostaining showed the clusters of TH-positive cells indicating presence of dopaminergic neurons[1] within the midbrain tissue. (H) These cells are GFP-positive indicating that they are of the implanted cell origin.

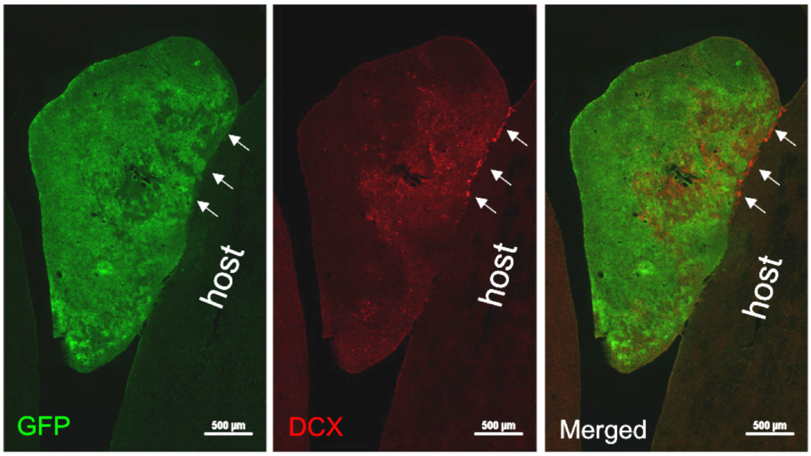

Supplementary Figure 11. Recruitment of DCX-expressing immature neurons from the host that persisted through a long period of 15 months after the implantation. Arrows indicate the presence of DCX-expressing- and GFP-negative cells at the interfaces of host lateral wall and the brain-like tissue.

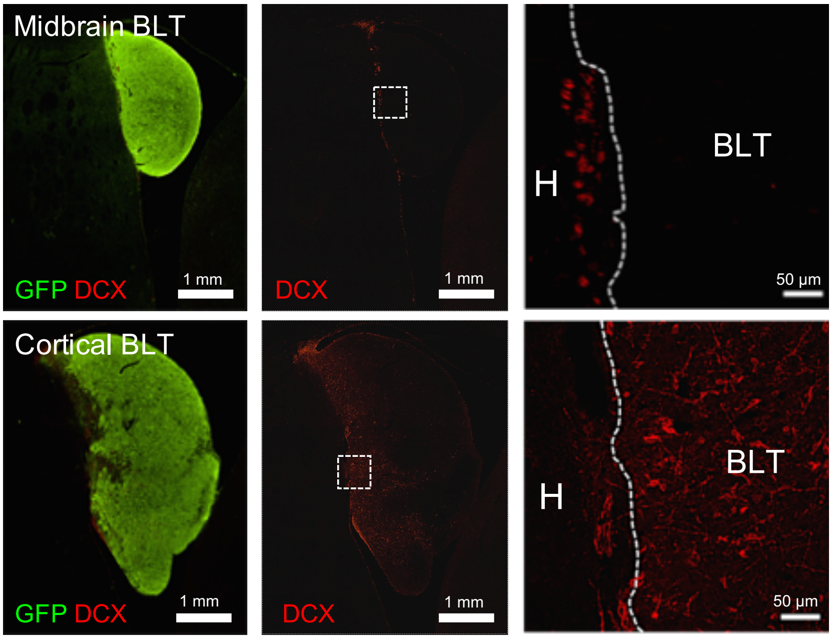

Supplementary Figure 12. Ability to recruit SVZ-born new neurons is specific to telencephalon-derived brain-like tissue. Immunostaining showed that there were no DCX-expressing cells that migrated into the tissue that was derived from midbrain precursor cells (top panels) unlike the telencephalon-derived tissue that extensively attract migration of DCX-expressing cells (bottom panels).

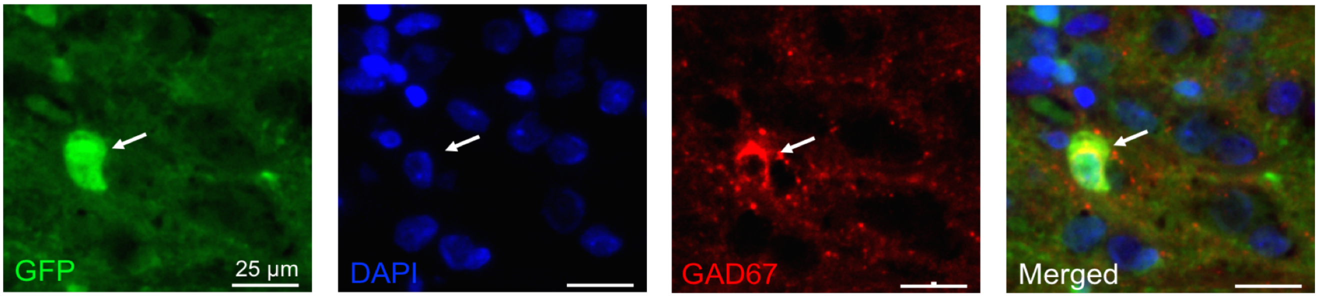

Supplementary Figure 13. Presence of GAD67-expressing GFP-positive neurons (arrows) in midbrain-derived tissue.

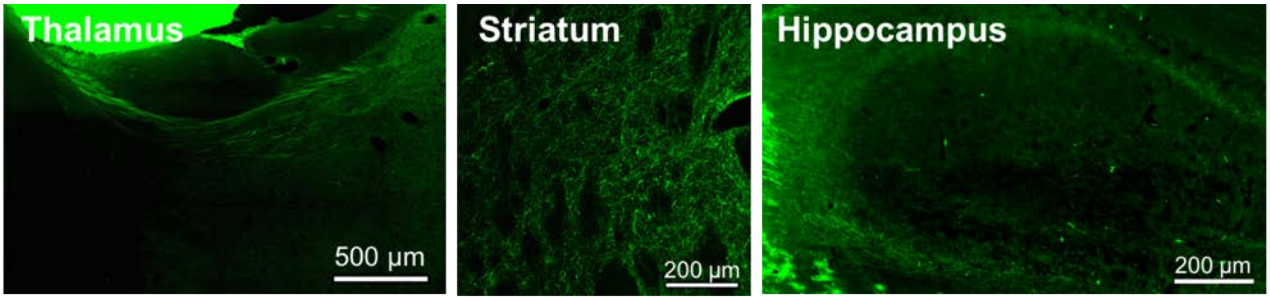

Supplementary Figure 14. The GFP-positive processes from the implants that projected into different areas of the host brain including thalamus, striatum, and CA3 region of hippocampus.

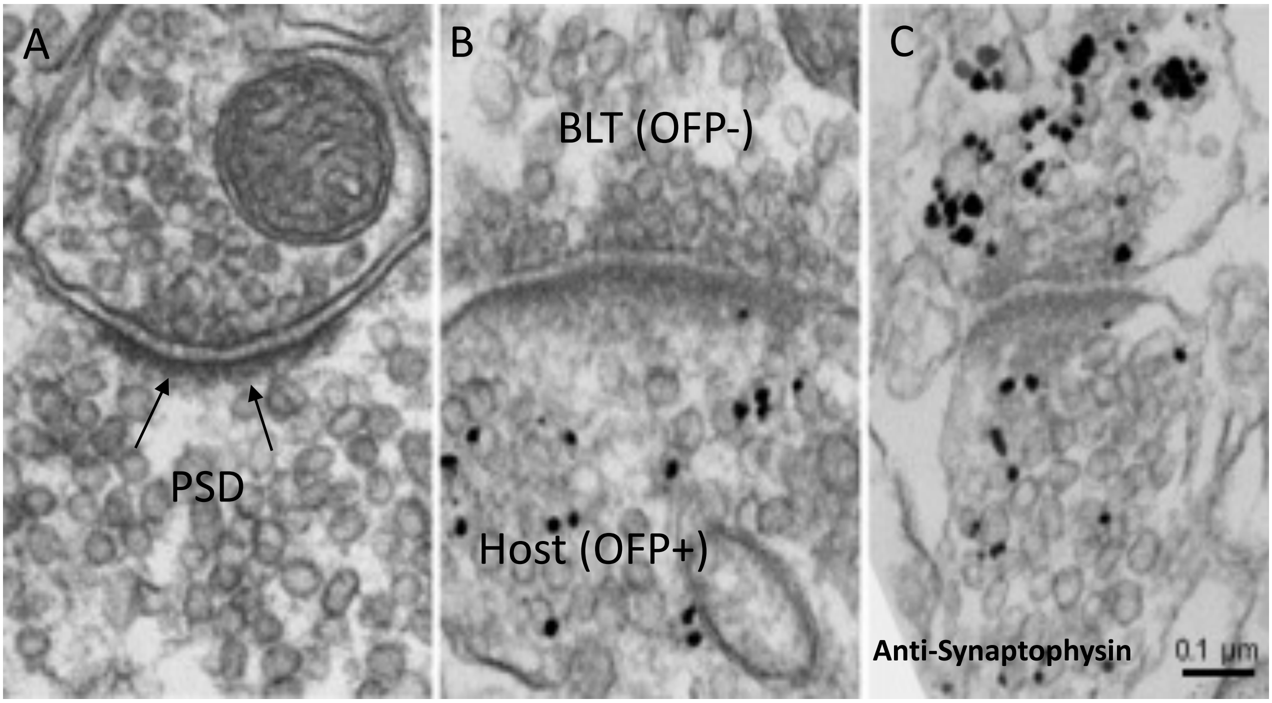

Supplementary Figure 15. Synapses between BLT neuron and the host interneurons. Atypical synapses between interneurons migrated from the host (labeled with OFP) and implanted neurons were prevalent in the BLT that was attached to striatum. (A) One striking feature of such atypical synapses is the cluster of synaptic vesicle-like structures near the postsynaptic density (PSD, marked by arrows). (B) Immunogold labeling demonstrated that the postsynaptic element is of the host origin (OFP-positive), and the presynaptic elements is from the BLT (OFP-negative). (C) The clusters of vesicles in the postsynaptic elements also labeled for synaptophysin, a synaptic vesicle marker. This finding is consistent with the fact that some GABAergic interneurons contain synaptic vesicles in their dendrites[2]

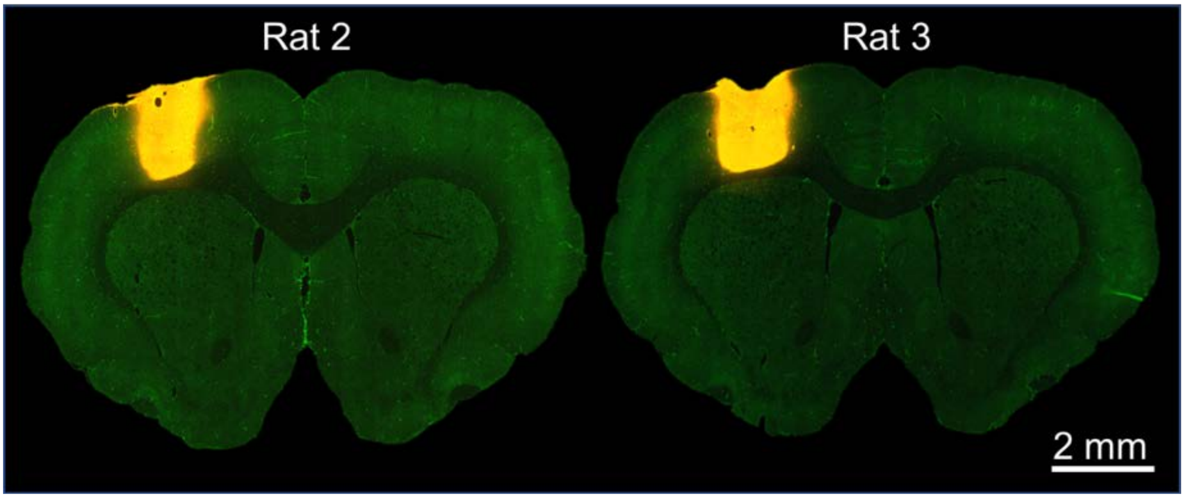

Supplementary Figure 16. Brain-like tissues that formed within the chronic cavities from the other two rats.

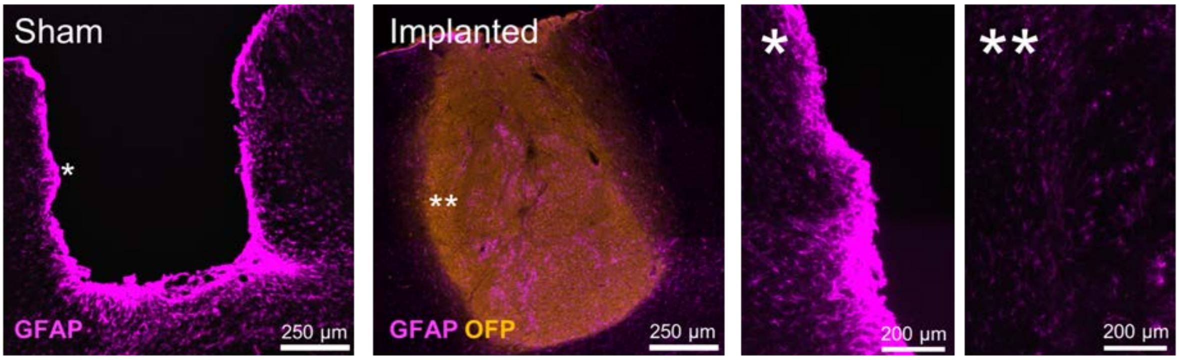

Supplementary Figure 17. Overview images of GFAP immunostaining in sham and implanted rat. * and ** represent the the area near the host border.

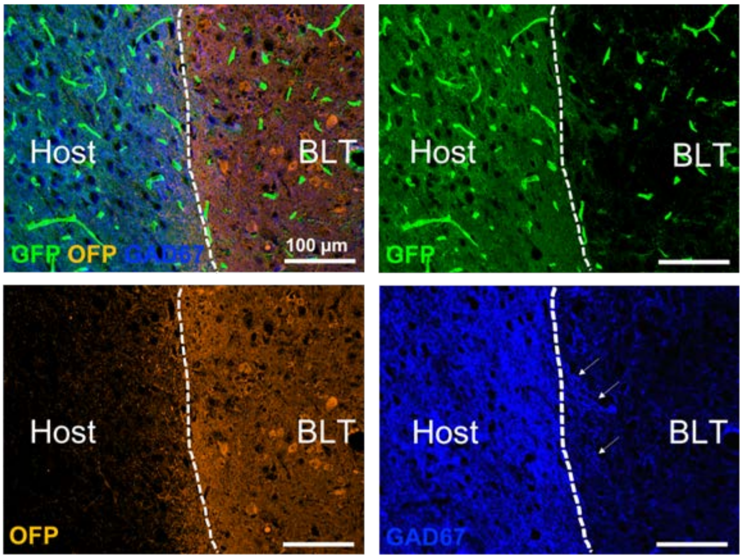

Supplementary Figure 18. Presence of GABAergic (GAD67-positive) processes (arrows) in the new formed tissue within the ablated cortex.

Supplementary notes

Supplementary note 1

Brain-like tissues from implantation of early midbrain precursor cells

We also tested whether early precursor cells from another brain region could develop into new tissue in the CSF. First, midbrain precursor cells from E14 embryos were isolated, enriched, and implanted into the CSF. However, there was no detectable new tissue in the ventricle. Therefore, it was tested whether the earlier embryonic stage of the midbrain could yield the formation of the new tissue. To this end, E11 midbrain precursor cells were used. Indeed, following implantation of E11 precursor cells, small clusters of cells could be detected by MRI within the ventricles 2 weeks after the implantation and were also detectable at 8 weeks (Supplementary Fig. 10A). The growth kinetics of the midbrain-derived tissue followed a similar pattern as that seen in cortical-derived tissue, but with a slower rate of expansion (Supplementary Fig. 10B) and smaller tissue size relative to the cortical-derived tissue (Supplementary Fig. 10C), implying a more limited proliferation capacity of the midbrain precursor cells. Immunohistochemistry results showed that all cell types were present (Supplementary Fig. 10D, Supplementary Fig. 10E, and Supplementary Fig. 10F). All neurons within the midbrain implant were of the implant origin suggesting there were no recruitment of new neurons from the host, unlike that found in the case of cortical implant. Furthermore, subset of neurons from the midbrain tissue expressed tyrosine hydroxylase (TH) a marker for dopaminergic neurons (Supplementary Fig. 10G, Supplementary Fig. 10H).

Supplementary References

[1] Margolis E. B., Coker A. R., Driscoll J. R., Lemaitre A. I. & Fields H. L. Reliability in the identification of midbrain dopamine neurons. PLoS ONE 5, e15222, doi:10.1371/journal.pone.0015222 (2010).

[2] Peters A., Palay S. L. & Webster H. D. *The fine structure of the nervous system : neurons and their supporting cells*. (Oxford University Press, 1991).
