# Supplementary material for: Neural precursor cells form integrated brain-like tissue when implanted into rat cerebrospinal fluid
Source: Commun Biol. 2018 Aug 14;1:114. doi: 10.1038/s42003-018-0113-8 (PMC6123740; doi:10.1038/s42003-018-0113-8)
Supplement: Supplementary file 2 — Description of additional supplementary items [file 42003_2018_113_MOESM2_ESM.docx]

**Supplementary Movies**

Supplementary Movie 1. 3D whole brain MRI image at 16-week post-implantation of E14 telencephalic neural precursor cells demonstrating the new tissue that occupied ventricle and CSF space within the host brain.

Supplementary Movie 2. Rats that had the brain-like tissue in the ventricles were kept more than one year after the implantation. These rats did not exhibit signs of distress and neurological symptoms
